# Predicting and explaining poor prognosis in diabetic kidney disease using SHAP-based interpretable machine learning

**DOI:** 10.1016/j.isci.2026.116280

**Published:** 2026-06-05

**Authors:** Man Qian, Lin Li, Yanli Cheng, Yue Hou, Shaojie Fu, Zhonggao Xu

**Affiliations:** 1 Department of Nephrology, The First Hospital of Jilin University, Changchun, China

**Keywords:** Health sciences, Nephrology, Artificial intelligence, Machine learning

## Abstract

Prognostic assessment of diabetic kidney disease (DKD) is essential for personalized management. This study developed eight machine learning models using data from 180 biopsy-proven patients with DKD to predict a composite endpoint of all-cause mortality, dialysis initiation, or renal transplantation. Internally, the Naive Bayes (NB) model achieved the highest accuracy of 82.3%, while the logistic regression (LR), support vector machine (SVM), and NB models shared the highest AUC of 0.788. An independent external validation confirmed robust generalizability, yielding an AUC of 0.834. SHAP analysis identified eGFR, serum albumin, C3, serum creatinine, and urinary red blood cell count (URBC) as the most impactful features. Feature stability was confirmed via a “leave-top1-out” sensitivity analysis. The models highlighted the predictive value of C3 and URBC by capturing non-linear patterns often missed by traditional linear methods, providing granular insights for personalized prognosis evaluation.

## Highlights

• Eight machine learning models predicted composite outcomes in biopsy-proven DKD
• External validation confirmed the models’ robust generalizability
• SHAP analysis highlighted five key predictors, notably serum C3 and urinary RBC
• Feature stability was rigorously validated via a Leave-Top1-Out strategy

## Graphical abstract

**Figure:**
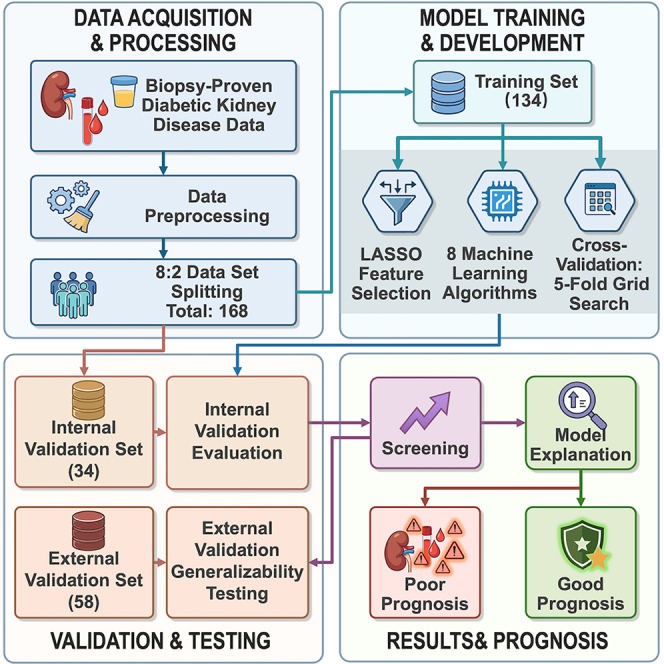

## Introduction

The global prevalence of diabetes is rapidly increasing, with projections estimating over 1.3 billion cases by 2050.^1^ Diabetic kidney disease (DKD) is a major complication affecting 30–40% of patients with diabetes and serves as a leading cause of end-stage renal disease (ESRD).^2^ While newer treatments such as renin-angiotensin-aldosterone system (RAAS) inhibitors, sodium-glucose cotransporter 2 inhibitors (SGLT2i), and glucagon-like peptide-1 receptor agonists (GLP-1RAs) have improved outcomes, many patients still experience a progressive decline in kidney function.^3^^,^^4^^,^^5^ Given that the presentation and progression of DKD vary significantly among individuals, identifying risk factors early is critical. Developing accurate predictive models is essential for guiding personalized treatment and reducing the overall burden of the disease.

Currently, standard management focuses on slowing progression rather than definitive cure. However, a major challenge is the lack of reliable markers to track disease progression and treatment effectiveness. The most common indicators, urinary protein and estimated glomerular filtration rate (eGFR), have significant limitations.^6^^,^^7^ Nearly half of patients with DKD do not have significant proteinuria, and several studies demonstrate no clear link between the rate of eGFR decline and mortality risk.^8^^,^^9^^,^^10^ These findings underscore the limitations of traditional markers and the complexity of DKD pathophysiology, suggesting that alternative factors contribute to poor outcomes.

Machine learning (ML) offers a powerful approach to address these challenges. As a subset of artificial intelligence, ML uses algorithms to identify complex patterns in data that traditional statistical methods may miss.^11^^,^^12^^,^^13^^,^^14^^,^^15^ In recent years, ML methodologies have been extensively applied across various medical disciplines, demonstrating robust performance in disease diagnosis and classification. Emerging studies indicate that optimized ML algorithms can achieve diagnostic accuracies ranging from 96% to 98.5% in identifying complex conditions such as lung cancer, brain tumors, and retinal diseases.^16^^,^^17^^,^^18^ Notably, recent findings have further validated the potential of ML in nephrology, with diagnostic accuracies reaching 94.03% for DKD and 97.9% for chronic kidney disease.^19^^,^^20^ Previous ML studies in DKD have successfully identified risk factors for disease progression with accuracies around 70–80%.^21^^,^^22^^,^^23^^,^^24^ However, many of these studies were limited by sparse datasets, high missing data rates, or a lack of pathological confirmation. Consequently, a highly accurate, biopsy-validated model for predicting DKD prognosis has not yet been established.

Leveraging a high-quality dataset from patients with biopsy-proven DKD and minimal missing data, this study addresses this gap. We retrospectively analyzed clinical and laboratory data to identify independent risk factors for poor prognosis. Subsequently, we developed and rigorously evaluated eight distinct ML models to predict composite outcomes. Using advanced interpretation techniques, we identified key predictive factors, including novel markers often overlooked by traditional statistics. Here, we demonstrate that integrating these biomarkers with interpretable ML models provides a robust, stable tool for stratifying patient risk, offering granular insights to guide the standardized and personalized management of DKD.

## Results

### Baseline characteristics

The baseline characteristics of the 180-patient cohort are presented in Table 1. The cohort was predominantly male (57.2%) with a median age of 53 years, and 51.7% presented with nodular sclerosing DKD.

**Table 1:** Baseline characteristics of patients

Variables	Numerical value
Gender Male Female	103 (57.2%) 77 (42.8%)
Age (y)	53 (43, 59)
Pathology Early Nodular sclerosing Diffuse sclerosing	77 (42.8%) 93 (51.7%) 10 (5.6%)
Age at diabetes diagnosis (y)	39 (32, 49)
Duration of diabetes (y)	10 (5, 15)
Hypertension No Grade 1 Grade 2 Grade 3	14 (7.8%) 4 (2.2%) 36 (20.0%) 115 (63.9%)
Duration of hypertension (m)	24 (3, 60)
FBG (mmol/L)	6.74 (5.24, 8.39)
HbA1c (%)	7.1 (6.2, 8.3)
Scr (μmol/L)	127.6 (90.6, 184.0)
eGFR (ml/min/1.73m^2^)	50.3 (31.1, 71.5)
Hb (g/L)	111 (97, 130)
Alb (g/L)	28.6 (±6.4)
24hUP (g/24h)	5.21 (2.77, 9.08)
MAU (mg/24h)	3987.19 (2039.46, 6176.55)
Follow-up results Composite endpoint No endpoint occurred Missing	79 (43.9%) 89 (49.4%) 12 (6.7%)

Categorical data are presented as frequencies and percentages. Continuous variables were presented as mean ± standard deviation for normally distributed data, while non-normally distributed data were shown as the median and interquartile range.

FBG, fasting blood glucose; HbA1c, glycated hemoglobin; Scr, serum creatinine; eGFR, estimated glomerular filtration rate; Hb, hemoglobin; Alb, serum albumin; 24hUP, 24-h urine protein; MAU, 24-h urinary microalbumin.

**Figure 1:**
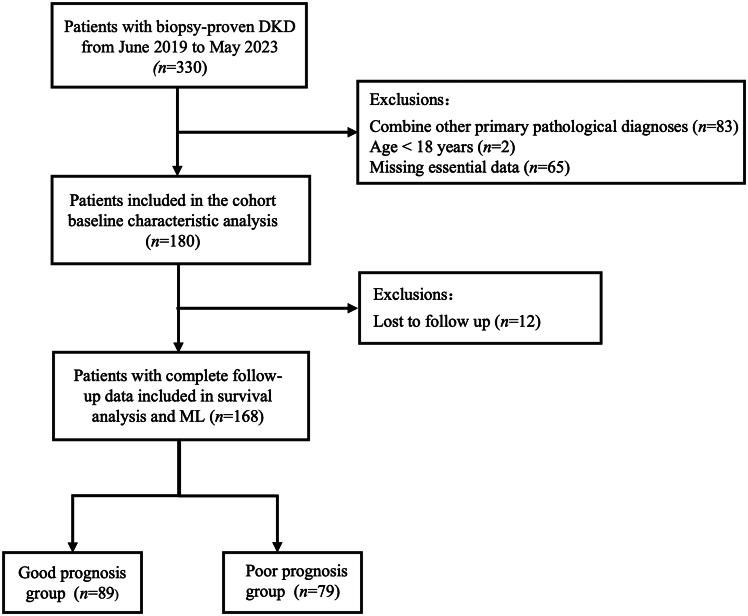
Flowchart of participant selection

Of the initial 180 patients, 12 were excluded due to loss of follow-up. The remaining 168 patients were stratified into a poor prognosis group (*n* = 79) and a good prognosis group (*n* = 89) (Figure 1). There was no significant difference in gender between the groups. However, significant differences were observed in the pathological stage of DKD *(p* < 0.001), and the prevalence of grade 3 hypertension, which was markedly higher in the poor prognosis group (*p* < 0.001). The two groups also exhibited distinct laboratory profiles. Compared to the good prognosis group, patients with a poor prognosis had significantly worse renal function [higher blood urea nitrogen (BUN) and serum creatinine (Scr), lower eGFR], more severe proteinuria, and elevated inflammatory markers [high-sensitivity C-reactive protein (CRP), erythrocyte sedimentation rate (ESR)]. Detailed baseline comparison is provided in Table 2.

**Table 2:** Characteristics of the poor prognosis group and good prognosis group patients

Variables	Poor prognosis group (*n* = 79)	Good prognosis group (*n* = 89)	*p* value
Gender (male, %)	43 (54.4)	53 (59.6)	0.503
Pathology, n(%) Early Nodular sclerosing Diffuse sclerosing	18 (22.8) 54 (68.4) 7 (8.9)	55 (61.8) 31 (34.8) 3 (3.4)	<0.001
Age (y)	52 (43, 60)	54 (45, 60)	0.466
BMI (kg/m^2^)	26.7 (24.5, 29.0)	25.7 (23.8, 27.9)	0.127
Age at diabetes diagnosis (y)	37.5 (32, 47)	41 (32, 50)	0.237
Duration of diabetes (y)	12 (5, 16)	10 (4.5, 15)	0.514
Duration of hypertension (m)	2 (0.25, 6)	2 (0.5, 5)	0.803
Hypertension, n(%) No Stage 1 Stage 2 Stage 3	9 (11.4) 1 (1.3) 10 (12.7) 59 (74.7)	13 (14.6) 2 (2.2) 25 (28.1) 49 (55.1)	<0.001
Hb (g/L)	102 (92, 119)	120 (107, 134)	<0.001
PLT (10^9^/L)	232 (185, 311)	245 (183, 291)	0.999
ChE (U/L)	8373 (6584, 9787)	9047 (7458, 10209)	0.090
Alb (g/L)	26.56 ± 5.19	30.89 ± 6.82	<0.001
BUN (mmol/L)	10.06 (7.66, 14.03)	7.48 (5.56, 9.38)	<0.001
Scr (μmol/L)	157.7 (114.9, 228.7)	101.3 (82.1, 135.0)	<0.001
eGFR (ml/min/1.73m^2^)	34.14 (23.10, 56.74)	64.38 (49.47, 82.30)	<0.001
UA (μmol/L)	416.09 ± 104.87	411.07 ± 94.07	0.745
FBG (mmol/L)	6.23 (4.86, 7.59)	6.83 (5.38, 9.30)	0.040
Cl (mmol/L)	107.63 ± 3.97	106.51 ± 3.31	0.048
TC (mmol/L)	5.78 (5.09, 7.39)	6.12 (4.79, 7.69)	0.960
TG (mmol/L)	1.92 (1.27, 2.52)	2.25 (1.44, 3.17)	0.109
HDL-C (mmol/L)	1.21 (1.04, 1.47)	1.18 (1.00, 1.49)	0.404
LDL-C (mmol/L)	3.62 (2.93, 4.49)	3.89 (2.93, 5.09)	0.387
Pi (mmol/L)	1.35 (1.20, 1.54)	1.31 (1.19, 1.41)	0.108
24hUV (L/24h)	1.8 (1.2, 2.3)	1.8 (1.5, 2.6)	0.177
24hUP (g/24h)	7.34 (4.29, 11.14)	3.74 (1.95, 6.22)	<0.001
MAU (mg/24h)	5216.62 (3609.81, 7066.37)	2795.83 (1467.75, 4839.10)	<0.001
24hU α1 microglobulin (mg/24h)	82.63 (48.99, 134.96)	36.70 (22.55, 65.50)	<0.001
24hU β2 microglobulin (mg/24h)	13.50 (4.15, 41.27)	1.57 (0.43, 7.92)	<0.001
24hUIgG (mg/24h)	653.87 (379.56, 981.76)	237.28 (126.87, 591.77)	<0.001
IgG (g/L)	6.64 (5.18, 9.43)	8.21 (6.58, 10.13)	0.011
IgA (g/L)	2.34 (1.78, 3.04)	2.43 (1.71, 3.28)	0.497
IgM (g/L)	0.78 (0.59, 1.09)	0.95 (0.71, 1.09)	0.059
C3 (g/L)	1.07 (0.90, 1.16)	1.11 (1.03, 1.21)	0.008
C4 (g/L)	0.33 (0.26, 0.41)	0.33 (0.27, 0.40)	0.674
CRP (mg/L)	3.14 (1.72, 7.45)	2.32 (1.35, 4.37)	0.001
HbA1c (%)	7.0 (6.0, 8.2)	7.1 (6.4, 8.2)	0.269
ESR (mm/h)	62 (42, 81)	35 (21, 60)	<0.001
TSH (μIU/mL)	2.95 (1.95, 5.02)	2.42 (1.53, 3.78)	0.037
FT3 (pmol/L)	3.34 (2.96, 3.71)	3.54 (3.22, 4.10)	0.009
FT4 (pmol/L)	11.57 (10.54, 13.12)	12.08 (11.14, 13.19)	0.111
URBC (/HPF)	4.9 (1.9, 10.4)	2.5 (1.3, 7.5)	0.042

Categorical data are presented as frequencies and percentages. Continuous variables were presented as mean ± standard deviation for normally distributed data, while non-normally distributed data were shown as the median and interquartile range.

Hb, hemoglobin; PLT, platelet count; ChE, serum cholinesterase; Alb, serum albumin; BUN, blood urea nitrogen; Scr, serum creatinine; eGFR, estimated glomerular filtration rate; UA, serum uric acid; FBG, fasting blood glucose; Cl, serum chloride; TC, total cholesterol; TG, triglycerides; HDL-C, high-density lipoprotein cholesterol; LDL-C, low-density lipoprotein cholesterol; Pi, serum phosphorus; 24hUV, 24-h urine volume; 24hUP, 24-h urine protein; MAU, 24-h urinary microalbumin; 24hU, 24-h urine; IgG, immunoglobulin G; IgA, immunoglobulin A; IgM, immunoglobulin M; C3: complements C3; C4, complements C4; CRP, high-sensitivity C-reactive protein; HbA1c, glycated hemoglobin; ESR, erythrocyte sedimentation rate; TSH, thyroid-stimulating hormone; FT3, free triiodothyronine; FT4, free thyroxine; URBC, urinary red blood cell count.

### Survival analysis

Among the 168 patients with complete follow-up data (median follow-up: 17.7 months), 79 experienced a composite endpoint event: 59 initiated regular dialysis, 2 received a renal transplant, and 18 died. The results of the univariate Cox regression analysis are detailed in Table 3.

**Table 3:** Univariate Cox regression analysis of prognostic risk factors for DKD

Variables	*HR* (95% *CI*)	*p* value
Gender (male)	0.797 (0.511, 1.242)	0.316
Pathology Early Nodular sclerosing Diffuse sclerosing	1 4.053 (2.337, 7.028) 4.986 (2.051, 12.021)	<0.001 <0.001
Age	0.996 (0.976, 1.016)	0.684
BMI	1.012 (0.956, 1.072)	0.677
Duration of diabetes	1.013 (0.987, 1.040)	0.335
Hypertension No Stage 1 Stage 2 Stage 3	1 1.876 (0.170, 20.742) 1.761 (0.384, 8.071) 4.237 (1.032, 17.393)	0.608 0.466 0.045
Hb	0.967 (0.956, 0.979)	<0.001
PLT	1.001 (0.998, 1.004)	0.463
ChE	1.000 (1.000, 1.000)	0.112
Alb	0.925 (0.896, 0.956)	<0.001
BUN	1.148 (1.092, 1.207)	<0.001
Scr	1.003 (1.002, 1.003)	<0.001
eGFR	0.964 (0.954, 0.974)	<0.001
UA	1.001 (0.999, 1.003)	0.480
FBG	0.932 (0.864, 1.005)	0.066
Cl	1.078 (1.010, 1.515)	0.025
TC	1.519 (0.910, 2.533)	0.110
TG	0.870 (0.732, 1.003)	0.112
HDL-C	0.837 (0.351, 1.997)	0.688
LDL-C	0.626 (0.349, 1.122)	0.115
Pi	1.833 (0.846, 3.974)	0.125
24hUV	0.752 (0.559, 1.011)	0.059
24hUP	1.082 (1.047, 1.119)	<0.001
MAU	1.000 (1.000, 1.000)	<0.001
24hU α1 microglobulin	1.005 (1.003, 1.007)	<0.001
24hU β2 microglobulin	1.014 (1.008, 1.020)	<0.001
24hUIgG	1.001 (1.000, 1.001)	<0.001
IgG	0.887 (0.817, 0.962)	0.004
IgA	0.903 (0.734, 1.112)	0.338
IgM	0.734 (0.461, 1.167)	0.191
C3	0.321 (0.108, 0.958)	0.041
C4	1.503 (0.195, 11.556)	0.696
CRP	1.007 (0.996, 1.018)	0.215
HbA1c	0.910 (0.773, 1.071)	0.256
ESR	1.017 (1.009, 1.025)	<0.001
TSH	1.053 (1.015, 1.093)	0.006
FT3	0.624 (0.466, 0.836)	0.002
FT4	0.894 (0.788, 1.014)	0.082
URBC	1.015 (1.006, 1.024)	0.001

**Figure 2:**
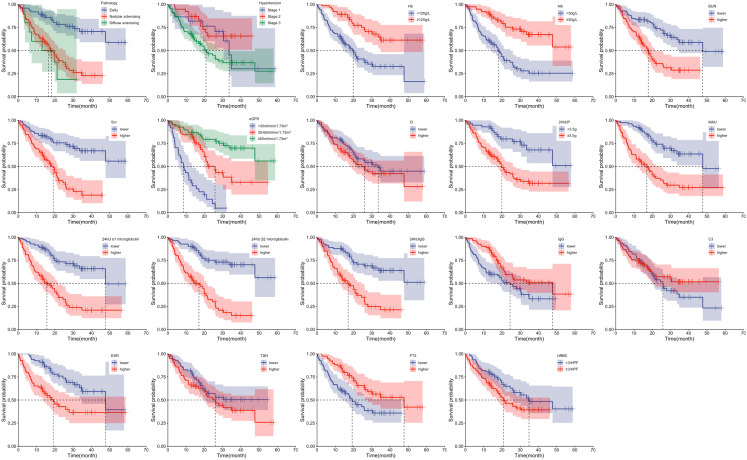
Kaplan-Meier survival curves for significant predictors from the univariate Cox regression analysis Hb, hemoglobin; Alb, serum albumin; BUN, blood urea nitrogen; Scr, serum creatinine; eGFR, estimated glomerular filtration rate; Cl, serum chloride; 24hUP, 24-h urine protein; MAU, 24-h urinary microalbumin; 24hU, 24-h urine; IgG, immunoglobulin G; C3, complements C3; ESR, erythrocyte sedimentation rate; TSH, thyroid-stimulating hormone; FT3, free triiodothyronine; URBC, urinary red blood cell count; lower, lower than the median; higher, above the median. The shaded areas represent the 95% confidence intervals.

Kaplan-Meier survival curves for the 19 significant continuous variables (converted to categorical variables) are shown in Figure 2. Multivariate Cox regression analysis subsequently identified four independent risk factors for poor prognosis: grade 3 hypertension (*HR* = 1.706, 95% *CI* 1.012–2.875, *p* = 0.045), lower serum albumin (Alb) (*HR* = 0.920, 95% *CI* 0.883–0.958, *p* < 0.001), lower eGFR (*HR* = 0.969, 95% *CI* 0.959–0.979, *p* < 0.001), and a higher urinary red blood cell count (URBC) (*HR* = 1.012, 95% *CI* 1.004–1.020, *p* = 0.003).

### Model development and validation

**Figure 3:**
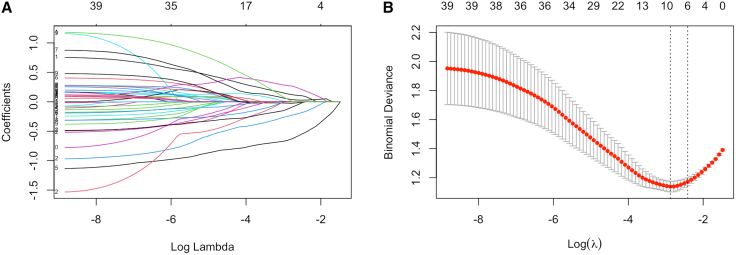
Results of the LASSO regression analysis (A) LASSO coefficient path plot for the 41 potential predictors. (B) LASSO cross-validation plot shows the optimal lambda value. Error bars in (B) represent the standard error (SE).

The dataset was randomly split into a training set (*n* = 134) and a validation set (*n* = 34). Using the training set exclusively, LASSO regression analysis screened the initial 41 predictors and identified nine key features at an optimal regularization parameter λmin = 0.05623 (Figure 3). The nine selected predictors were pathological results, hemoglobin (Hb), Alb, Scr, eGFR, 24-h urinary microalbumin (MAU), α1-microglobulin, complement C3, and URBC. Collinearity diagnosis confirmed that the variance inflation factor (VIF) values for all selected variables were below 5.

**Figure 4:**
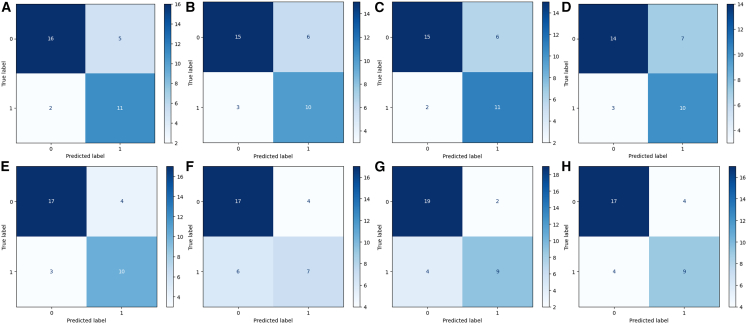
Confusion matrices for each machine learning model on the validation set (A) Logistic regression, (B) random forest, (C) support vector machine, (D) K-nearest neighbors, (E) gradient boosting decision tree, (F) adaptive boosting, (G) Naive Bayes, and (H) extreme gradient boosting.

**Figure 5:**
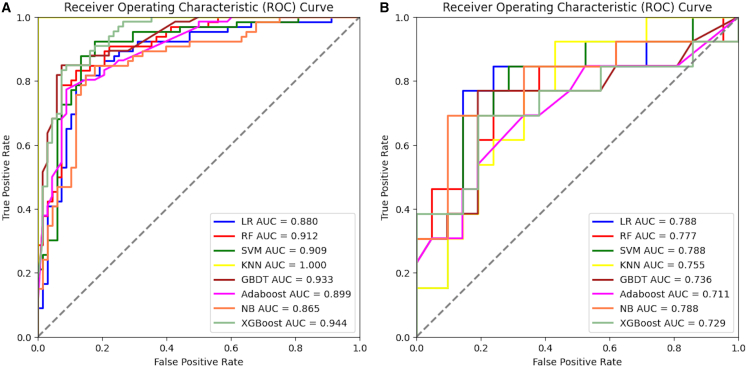
Receiver operating characteristic (ROC) curves of the eight models LR, Logistic regression; RF, random forest; SVM, support vector machine; KNN, K-nearest neighbors; GBDT, gradient boosting decision tree; AdaBoost, adaptive boosting; NB, Naive Bayes; XGBoost, extreme gradient boosting; and AUC, area under the curve.

**Figure 6:**
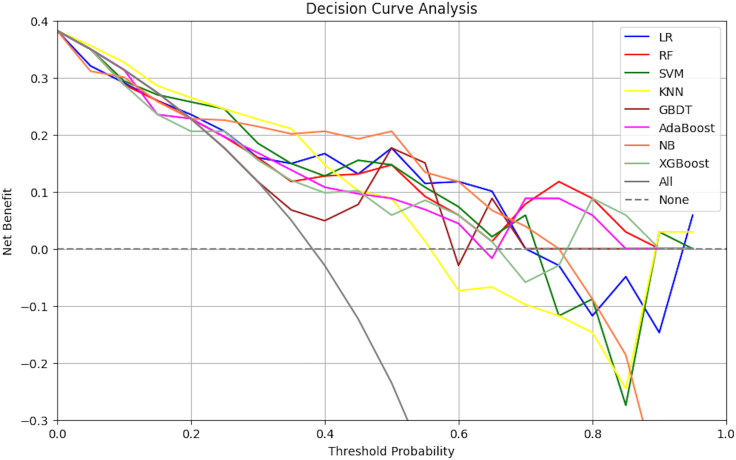
Decision curve analysis of the eight models LR, logistic regression; RF, random forest; SVM, support vector machine; KNN, K-nearest neighbors; GBDT, gradient boosting decision tree; AdaBoost, adaptive boosting; NB, Naive Bayes; XGBoost, extreme gradient boosting.

The confusion matrices for each model’s performance on the validation set are shown in Figure 4. The accuracy, precision, recall, F1-score, and area under the curve (AUC) for all models are summarized in Table 4. All eight models achieved an accuracy greater than 70%. The Naive Bayes (NB) model yielded the highest accuracy (82.3%), followed by logistic regression (LR) and gradient boosting decision tree (GBDT) (both 79.4%). The LR model achieved the highest F1-score (0.759). Receiver operating characteristic (ROC) curve analysis (Figure 5) showed that the LR, support vector machine (SVM), and NB models achieved the highest AUC of 0.788 (LR 95% *CI*, 0.601–0.949; SVM 95% *CI*, 0.615–0.938; NB 95% *CI*, 0.604–0.949) on the validation set. The random forest (RF) model achieved an AUC of 0.777 (95% *CI*, 0.593–0.934). Notably, the K-nearest neighbor (KNN) model demonstrated an AUC of 1.0 on the training set but dropped to 0.755 on the validation set. The decision curve analysis (DCA) indicated that the LR and NB models provided the highest net benefit across a broad range of threshold probabilities (Figure 6). Based on these metrics, the LR, RF, SVM, and NB models were selected as the most promising for this prediction task.

**Table 4:** The comparative assessment of performance metrics for internal validation among eight models

Model	Accuracy-training set	Accuracy -validation set	Precision	Recall	F1-score	AUC-training set	AUC-validation set
LR	0.813	0.794	0.688	0.846	0.759	0.880	0.788
RF	0.851	0.735	0.625	0.769	0.690	0.912	0.777
SVM	0.866	0.765	0.647	0.846	0.733	0.909	0.788
KNN	1.000	0.706	0.588	0.769	0.667	1.000	0.755
GBDT	0.873	0.794	0.714	0.769	0.741	0.933	0.736
AdaBoost	0.843	0.706	0.636	0.538	0.583	0.899	0.711
NB	0.762	0.823	0.818	0.692	0.750	0.865	0.788
XGBoost	0.873	0.765	0.692	0.692	0.692	0.944	0.729

LR, logistic regression; RF, random forest; SVM, support vector machine; KNN, K-nearest neighbors; GBDT, gradient boosting decision tree; AdaBoost, adaptive boosting; NB, Naive Bayes; XGBoost, extreme gradient boosting; AUC, area under the curve.

### External validation

**Figure 10:**
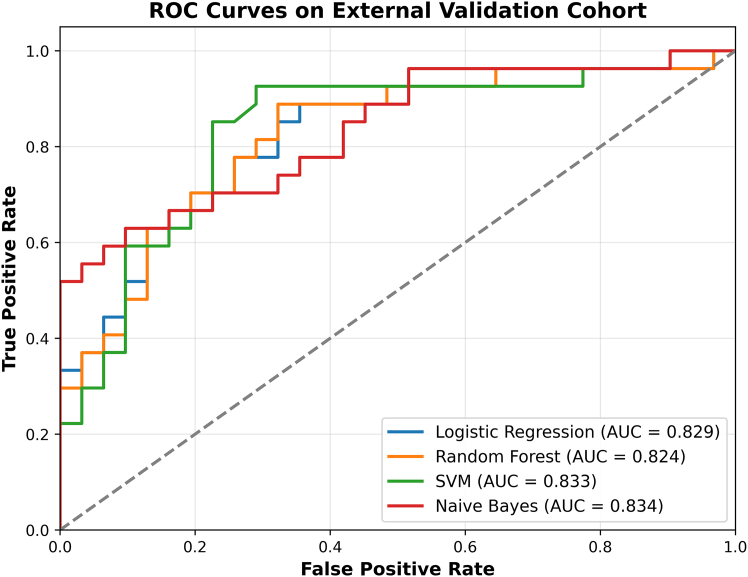
ROC Curves on the external validation cohort

External validation was performed on an independent dataset of 58 patients to rigorously assess the models’ clinical applicability. This validation tests the models’ ability to generalize to patient populations that were not involved in the model training or internal validation processes. Table S1 describes the baseline characteristics of the patients in the external validation set compared to those in the model development cohort. While the cohorts were largely comparable, statistically significant differences were observed in hypertension grading, pathological classification, and serum C3 levels. The four optimized ML models were directly evaluated on this external dataset without retraining. As detailed in Table S2, all models demonstrated robust discriminative ability. The NB model achieved the highest AUC of 0.834 (Figure 10, 95% *CI*, 0.713–0.929), followed closely by the SVM model (AUC = 0.833, 95% *CI*, 0.714–0.935), LR model (AUC = 0.829, 95% *CI*, 0.720–0.928), and RF model (AUC = 0.824, 95% *CI*, 0.712–0.931). Notably, the AUCs obtained on the external validation set were higher than those obtained on the internal validation set. These findings demonstrate the strong generalizability and robustness of the established models on unseen data, even in the presence of baseline clinical variations.

### Explanation of risk factors

**Figure 7:**
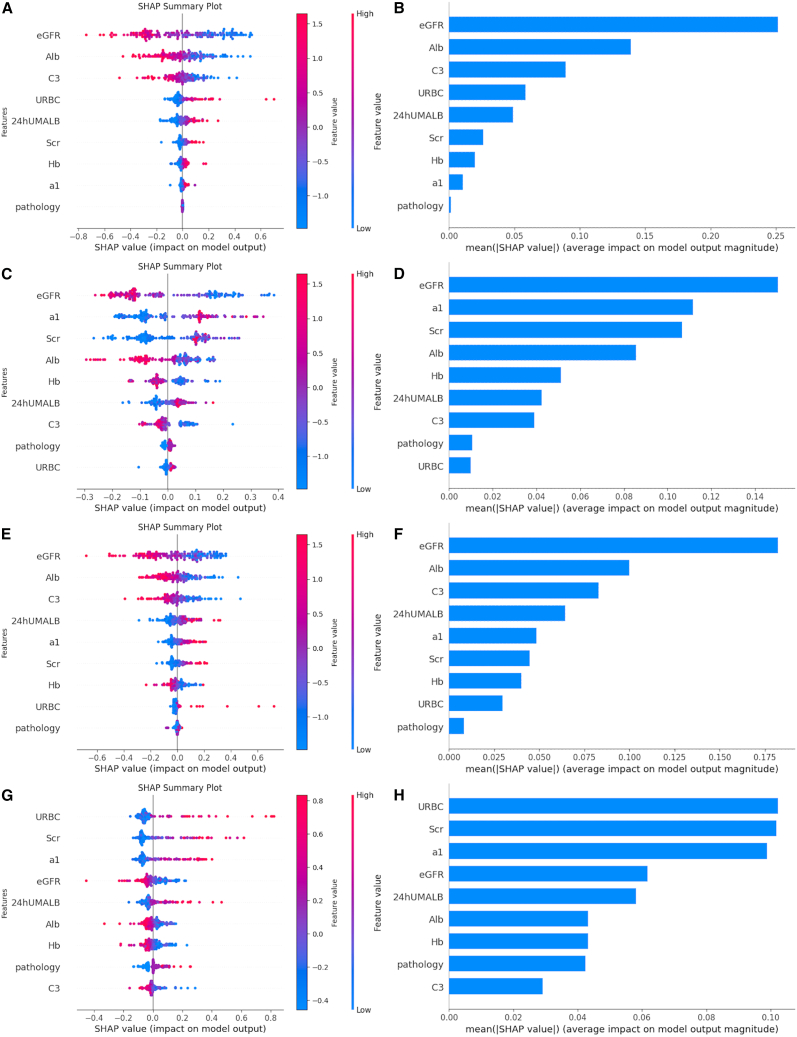
SHAP visualization of the four models (A) SHAP global bee swarm plot for the logistic regression model; (B) SHAP global bar plot for the logistic regression model; (C) SHAP global bee swarm plot for the Random Forest model; (D) SHAP global bar plot for the Random Forest model; (E) SHAP global bee swarm plot for the support vector machine model; (F) SHAP global bar plot for the support vector machine model; (G) SHAP global bee swarm plot for the Naive Bayes model; (H) SHAP global bar plot for the Naive Bayes model. Hb, hemoglobin; Alb, serum albumin; Scr, serum creatinine; eGFR, estimated glomerular filtration rate; 24hUMALB, 24-h urinary microalbumin; a1, 24-h urinary α1-microglobulin; C3, complement C3; URBC, urinary red blood cell count.

SHapley Additive exPlanations (SHAP) analysis was applied to the four best-performing models (Figure 7). In the LR model, lower levels of eGFR, Alb, and C3 were the primary contributors to predicting an endpoint event. In the RF model, eGFR, 24-h urinary α1-microglobulin, and Scr were the top predictors, with higher α1-microglobulin levels associated with poor prognosis. The SVM model showed feature importance patterns similar to the LR model. In contrast, the NB model identified URBC, Scr, and 24-h urinary α1-microglobulin as the top three predictors. C3 exhibited a lower mean SHAP value in the NB model compared to the other algorithms.

### Reliability and stability assessment of feature importance

**Figure 8:**
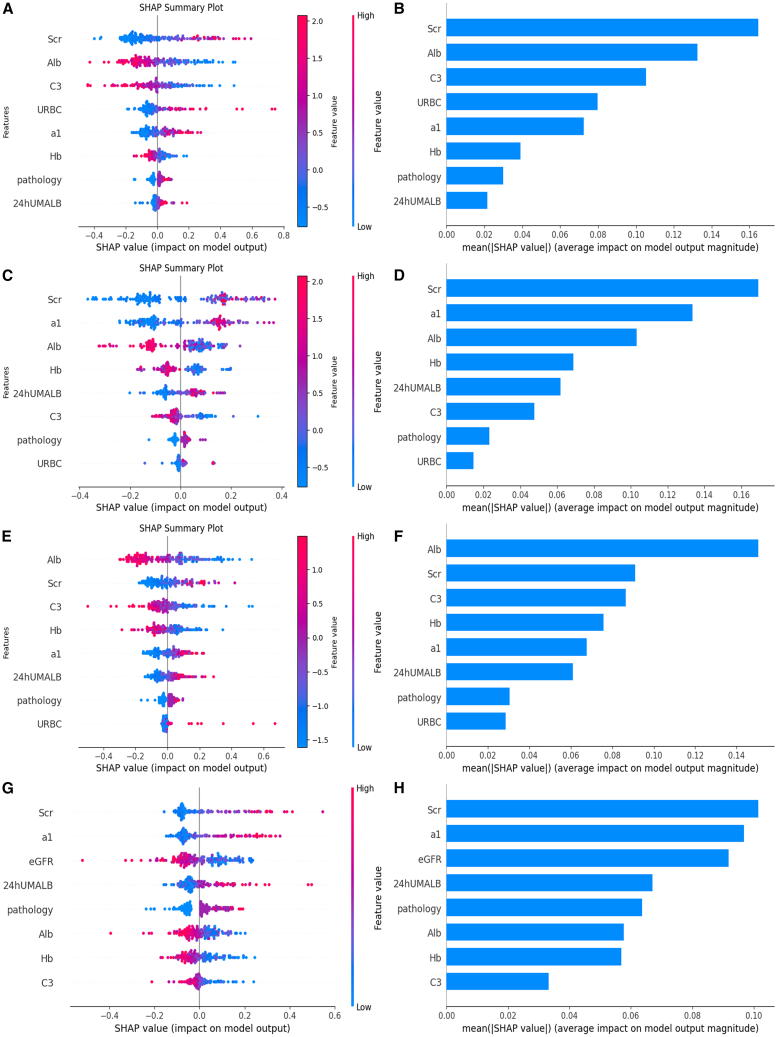
SHAP results of the “leave-top1-out” analysis (A) SHAP global bee swarm plot for the logistic regression model; (B) SHAP global bar plot for the logistic regression model; (C) SHAP global bee swarm plot for the random forest model; (D) SHAP global bar plot for the random forest model; (E) SHAP global bee swarm plot for the support vector machine model; (F) SHAP global bar plot for the support vector machine model; (G) SHAP global bee swarm plot for the Naive Bayes model; (H) SHAP global bar plot for the Naive Bayes model.

To assess feature robustness, a “leave-top1-out” analysis was performed (Figure 8). In the initial LR, RF, and SVM models, eGFR was the top-ranked feature. Upon the exclusion of eGFR and model retraining, SHAP analysis revealed that Scr consistently replaced eGFR as the top-ranked predictor. The rankings of the remaining features (specifically Alb and C3) remained stable. Similarly, in the NB model, removing the top feature (URBC) did not alter the relative importance of the remaining predictors, with Scr and α1-microglobulin maintaining high rankings.

**Figure 9:**
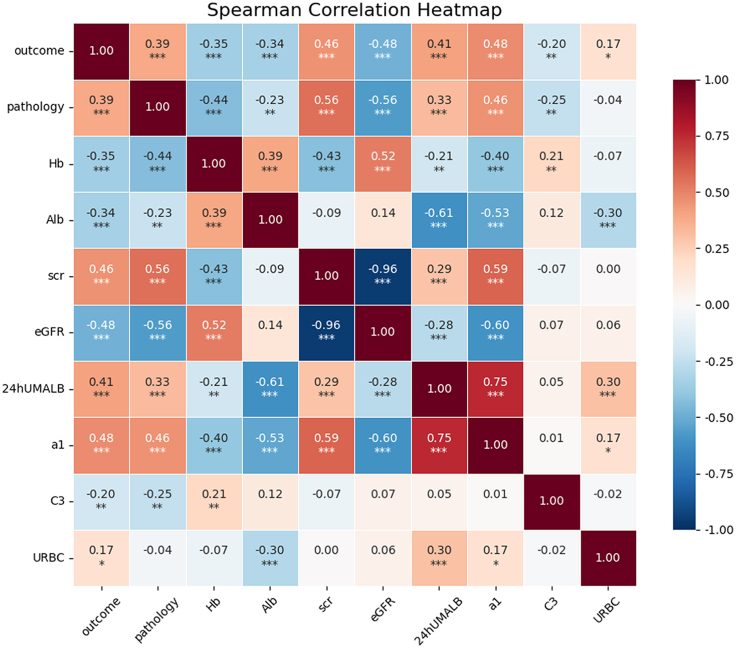
Heatmap of Spearman’s rank correlation analysis Outcome was defined as all-cause mortality, initiation of regular dialysis, or receiving a renal transplant. Asterisks indicate significance levels (∗∗∗*p* < 0.001, ∗∗*p* < 0.01, and ∗*p* < 0.05).

Spearman’s rank correlation analysis (Figure 9) confirmed that all features included in the models were significantly correlated with the clinical outcome (*p* < 0.05). eGFR (*r* = −0.48, *p* < 0.001), 24-h urinary α1-microglobulin (*r* = 0.48, *p* < 0.001) and Scr (*r* = 0.46, *p* < 0.001) exhibited the strongest correlations. Features such as C3 and URBC showed statistically significant but lower linear correlation coefficients compared to the renal function markers.

## Discussion

This study successfully identified independent predictors of poor prognosis in biopsy-proven DKD using traditional survival analysis and subsequently developed high-performing, interpretable ML models. Our Cox regression analysis confirmed that grade 3 hypertension, lower Alb, lower eGFR, and higher URBC are independent risk factors. Complementing this, SHAP analysis of the ML models validated the predictive importance of eGFR and Alb and highlighted the critical roles of URBC and serum C3, which may be underestimated by conventional linear methods. Crucially, to address the common criticism of black box models, we implemented a rigorous “leave-top1-out” strategy and validated the models in an independent external cohort, which confirmed the stability, physiological validity, and strong generalizability of our feature selection.

Our study cohort comprised 180 patients with a biopsy-proven diagnosis of DKD, offering a higher diagnostic certainty than studies relying solely on clinical criteria. The cohort was predominantly male (57.2%), contrasting with some epidemiological findings suggesting a female preponderance in DKD.^25^ This male predominance may reflect a higher burden of risk factors such as smoking and obesity in men,^26^ which accelerate renal insufficiency. While diabetes duration was comparable to other studies, our patients exhibited a notably higher prevalence of hypertension and renal insufficiency.^27^^,^^28^^,^^29^ We attribute this to selection bias inherent in a biopsy-based cohort: patients undergoing renal biopsy typically present with atypical features or more severe disease progression than the general diabetic population. Regarding proteinuria, the patterns observed here differ from some cross-sectional reports,^30^^,^^31^ likely due to the widespread use of medications such as RAAS inhibitors, SGLT2i, and GLP-1RA, which mitigate proteinuria levels even in advanced disease.

Patients with a poor prognosis exhibited greater severity of glomerular lesions and more advanced hypertension. This aligns with previous multicenter studies linking systolic blood pressure and nodular lesions to rapid renal decline.^32^ The mechanism involves intraglomerular hypertension, RAAS activation, and endothelial damage caused by chronic hypertension, underscoring the need for combining strict blood pressure control with renal protection strategies. Notably, we observed lower fasting blood glucose (FBG) levels in the poor prognosis group, contradicting the consensus that hyperglycemia drives DKD.^33^^,^^34^ As suggested by the counterintuitive data, this likely reflects reduced insulin clearance, which is a hallmark of advanced renal failure where the renal degradation of insulin diminishes, prolonging its half-life and spontaneously lowering blood glucose.^35^ Additionally, higher serum chloride levels in the poor prognosis group likely stem from unmeasured confounding factors such as diuretic usage or dietary intake, highlighting a need for further controlled investigation.

Hypoalbuminemia and eGFR decline are well-established prognostic markers.^36^^,^^37^^,^^38^^,^^39^ Our findings reinforce their importance but also highlight the value of non-traditional markers. Previous research has linked anemia and thyroid dysfunction [low free triiodothyronine (FT3)] to poor outcomes in DKD.^40^^,^^41^^,^^42^^,^^43^ Our univariate analysis supports these associations, suggesting that metabolic disturbances and tissue hypoxia may impair peripheral thyroid hormone conversion, potentially opening avenues for thyroid replacement therapy as a protective strategy.

A key contribution of this study is the granular analysis of URBC and the identification of C3 as a predictor. While prior studies treated hematuria as a binary variable (present or absent),^44^^,^^45^ our ML models utilized URBC as a continuous variable, capturing the severity of glomerular injury more precisely. Furthermore, SHAP analysis identified lower serum C3 as a significant predictor of poor prognosis. This finding aligns with emerging evidence implicating the complement system in DKD pathogenesis.^46^ Complement activation via the alternative and lectin pathways drives renal inflammation and fibrosis. The lower serum C3 levels observed likely reflect the consumption of complement components due to their activation and intra-renal deposition, an immune-mediated process that accelerates progression to ESRD. Importantly, while Spearman analysis showed lower linear correlations for C3 and URBC compared to eGFR, the ML models assigned them high importance. This discrepancy suggests that these markers may influence prognosis through non-linear threshold effects that traditional statistics fail to capture, demonstrating the unique value of ML in uncovering complex biological signals.

We developed eight ML models, with LR, SVM, and NB achieving the highest robust performance (AUC = 0.788 in internal validation). Feature engineering using LASSO regression was crucial for mitigating overfitting, given our moderate sample size. Our models achieved performance comparable to larger cohorts,^47^^,^^48^ suggesting that the high-quality, biopsy-proven nature of our data provided a strong signal-to-noise ratio. While ML models often outperform traditional methods, their “black box” nature can limit clinical interpretation. To address this, the SHAP method, as introduced by Lundberg et al.,^49^ offers a unified framework. It provides a consistent and computationally efficient means of decomposing model predictions into additive feature contributions.^50^ However, it is crucial to recognize the fundamental principles and limitations of supervised models in this context. Supervised models possess two distinct types of accuracy: target prediction accuracy and feature importance accuracy. While target prediction accuracy can be validated against ground truth labels, feature importance lacks a corresponding ground truth for validation, indicating the absence of ground truth in feature assessment. Feature importance derived from supervised models refers to contributions to prediction rather than true associations. Consequently, the function of an explainer such as SHAP indicates that SHAP is forced to exclusively rely on a given supervised model with unvalidated features, faithfully inheriting and potentially amplifying biases in feature importance from the model. To validate true associations and overcome these inherent biases, two mandatory key components must be examined: consistency and dose-response relationships. Furthermore, ordered sets of features must be examined instead of non-ordered sets for reliable feature assessment. We strictly adhered to these principles in our study. First, to assess consistency within ordered sets of features, we employed the “leave-top1-out” validation strategy. A critical finding was that when the top predictor (eGFR) was removed, the models consistently substituted it with Scr. This substitution is physiologically and mathematically expected, as eGFR is calculated based on Scr levels. The fact that the models immediately identified the next best proxy for renal filtration confirms that they learned the underlying pathophysiology rather than relying on data artifacts. Meanwhile, the ordered rankings of other features (Alb, C3) remained stable, demonstrating robust consistency. Second, to validate dose-response relationships independently of label-driven model errors, we utilized a dual approach. Visually, the SHAP summary plots demonstrated a clear gradient effect. Statistically, we utilized Spearman’s rank correlation analysis. This confirmed that our key predictors exhibited significant monotonic biological gradients (dose-response) with clinical prognosis, ensuring a reliable feature assessment.

A major strength of our study is the successful validation of the predictive models in an independent external cohort. Our models, particularly the NB and SVM algorithms, demonstrated exceptional prognostic performance on this unseen data, achieving AUCs ranging from 0.824 to 0.834. Interestingly, the models exhibited even higher discriminative performance on the external dataset compared to the internal validation set. In clinical prediction modeling, this phenomenon can largely be attributed to differences in patient case-mix (spectrum effect). The external cohort presented significant variations in baseline characteristics, such as hypertension grading and C3 levels. A more polarized distribution of these clinical features in the external cohort mathematically simplifies the binary classification task, thereby contributing to a higher AUC. Furthermore, the relatively small size of our internal validation set (*n* = 34) may have yielded a more conservative and statistically variable AUC estimate. Importantly, the widely overlapping 95% *CI* between the two cohorts confirms that the model’s fundamental discriminative ability remains robust and consistent across different clinical settings. The robust discriminative power across geographically distinct cohorts confirms that the algorithms successfully captured intrinsic, generalizable biological patterns underlying DKD progression rather than cohort-specific noise. Ultimately, the stability of rank-independent metrics (AUC) demonstrates the models’ reliable patient-ranking capability and robust adaptability in diverse clinical settings with varying baseline risks.

Our study demonstrates that integrating traditional clinical markers with interpretable ML significantly enhances prognostic assessment in DKD. Beyond standard risk factors such as hypertension and hypoalbuminemia, our models uniquely highlighted the predictive value of URBC and serum C3, offering granular insights into complement activation and glomerular injury. By validating feature stability through rigorous sensitivity analysis and demonstrating strong generalizability via external validation, we provide a robust framework for stratifying high-risk patients. Future work will focus on validating these models in large-scale, international multi-center prospective cohorts and integrating multi-omics data to further elucidate the biological mechanisms driving DKD progression.

### Limitations of the study

This study has several limitations. First, the sample size was moderate, which may have constrained the predictive power of more complex, non-linear algorithms such as extreme gradient boosting (XGBoost) compared to simpler models. Second, our study population was restricted to a single ethnicity. Thus, the broad generalizability of our models to ethnically and globally diverse populations remains to be prospectively verified. Finally, due to the retrospective design, we could not fully account for post-biopsy treatment regimens, which are potential confounding factors influencing disease progression.

## Resource availability

### Lead contact

Further information and requests for resources should be directed to and will be fulfilled by the lead contact, Zhonggao Xu (zhonggao@jlu.edu.cn).

### Materials availability

This study did not generate new unique reagents.

### Data and code availability

• Data will be made available from the lead contact on request.
• All original code has been deposited at GitHub and is publicly accessible from the publication date. The web link is listed in the key resources table.
• Any additional information required to reanalyze the data reported in this paper is available from the lead contact upon request.

## Acknowledgments

This study was supported by the 10.13039/501100001809National Natural Science Foundation of China (grant no. 82570845 and 82300854), the 10.13039/100007847Natural Science Foundation of Jilin Province (grant no. YDZJ202401437ZYTS), and the Jilin University Bethune Program Project (grant no. 2025B29). We also thank Meihe Hospital of The First Hospital of Jilin University for their support with the external validation data.

## Author contributions

M.Q. drafted the original manuscript, revised the manuscript, created visualizations, performed validation, designed methodology, conducted investigation, curated data, and conceptualized the study. L.L. revised the manuscript, conducted an investigation, and curated data. Y.C. revised the manuscript, performed validation, and conducted formal analysis. Y.H. revised the manuscript, created visualizations, and curated data. S.F. revised the manuscript, provided supervision, designed methodology, and conceptualized the study. Z.X. revised the manuscript, provided supervision, designed methodology, conceptualized the study, managed project administration, and acquired funding. All authors interpreted the data, revised the manuscript, approved the final content, and read and approved the final manuscript. All authors contributed to the article and approved the submitted version.

## Declaration of interests

The authors declare no conflicts of interest.

## STAR★METHODS

### Key resources table

**Table:** 

REAGENT or RESOURCE	SOURCE	IDENTIFIER
**Deposited data**		

Source Code	This paper	https://github.com/qianman-111/diabetic-kidney-disease

**Software and algorithms**		

SPSS 29.0	IBM	https://www.ibm.com/products/spss-statistics
R 4.2.3	The R Foundation	https://www.r-project.org
Python 3.12	Python Software Foundation	https://www.python.org

### Experimental model and study participant details

#### Human subjects

We retrospectively enrolled 180 patients who were pathologically diagnosed with DKD following a renal biopsy at the Department of Nephrology, The First Hospital of Jilin University, between June 2019 and May 2023. The study cohort consisted of 103 males (57.2%) and 77 females (42.8%), with a median age of 53 years. The inclusion criteria were as follows: (1) a previously confirmed diagnosis of diabetes; (2) age ≥18 years; (3) a pathological diagnosis consistent with the 2010 Renal Pathology Society (RPS) classification criteria for DKD; (4) a follow-up duration of ≥12 months or the occurrence of an endpoint event within 12 months. The exclusion criteria were: (1) missing data for key baseline variables required for analysis, such as diabetes duration or hypertension status; (2) a primary pathological diagnosis other than DKD, or unclear evidence of coexisting DKD; (3) follow-up of less than 12 months or loss to follow-up. See Figure 1 for the detailed flowchart. The diagnosis of diabetes was based on the criteria of the American Diabetes Association. The eGFR was calculated using the Chronic Kidney Disease Epidemiology Collaboration (CKD-EPI) equation.

To rigorously assess the generalizability and clinical applicability of the developed machine learning models, an independent external validation cohort (*n* = 58) was retrospectively established. These patients were recruited from Meihe Hospital of The First Hospital of Jilin University, a regional medical facility located approximately 200 km away from the primary study center. Patient screening strictly adhered to the identical inclusion and exclusion criteria used for the primary model development cohort.

#### Ethics approval and security

This study was reviewed and approved by the Ethics Committee of The First Hospital of Jilin University (Approval No. 2025-005). To ensure patient privacy and data security, all personal identification information was anonymized prior to analysis.

### Method details

#### Clinical and laboratory data collection

We collected patient history, including age, sex, height, weight, Body Mass Index (BMI), age at diabetes diagnosis, duration of diabetes, age at hypertension diagnosis, duration of hypertension, and hypertension grade. The duration of diabetes and hypertension was the interval from initial diagnosis to renal biopsy.

Renal biopsy pathology reports and the last laboratory test results available before the biopsy were collected. These included: hemoglobin (Hb), platelet count (PLT), serum cholinesterase (ChE), serum albumin (Alb), blood urea nitrogen (BUN), serum creatinine (Scr), eGFR, serum uric acid (UA), fasting blood glucose (FBG), serum chloride (Cl), total cholesterol (TC), triglycerides (TG), high-density lipoprotein cholesterol (HDL-C), low-density lipoprotein cholesterol (LDL-C), serum phosphorus (Pi), 24-h urine volume (24hUV), 24-h urine protein (24hUP), 24-h urinary microalbumin (MAU), α1 and β2 microglobulin, and immunoglobulin G (IgG). Additional blood tests included serum immunoglobulins (IgG, IgA, IgM), complements C3 and C4, high-sensitivity C-reactive protein (CRP), glycated hemoglobin (HbA1c), erythrocyte sedimentation rate (ESR), thyroid-stimulating hormone (TSH), free triiodothyronine (FT3), free thyroxine (FT4), and urinary red blood cell count (URBC).

#### Follow-up and outcome definition

Follow-up records were collected from the Electronic Medical Record (EMR) system. Patients with no or insufficient follow-up records were contacted by telephone to confirm outcomes by May 2024. The composite endpoint (poor prognosis) was defined as all-cause mortality, initiation of regular dialysis, or receiving a renal transplant. If a patient experienced multiple endpoint events, the first event and its corresponding date were recorded.

#### Data preprocessing

Missing data were addressed first: variables with >10% missing data were excluded; continuous variables with <10% missing values were imputed using multiple imputation. Categorical variables had complete capture. The dataset was manually reviewed to remove clear outliers. All continuous variables were standardized using the StandardScaler function from the “scikit-learn” library in Python. To prevent data leakage, continuous variables in the external validation cohort were standardized using the exact same scaler fitted on the primary training set.

#### Model development

The final dataset of 168 patients was randomly divided into a training set (80%) and an independent internal validation set (20%). To reduce multicollinearity and prevent overfitting, LASSO regression was used for feature selection on the training set. Eight machine learning models were developed using Python 3.12: Logistic Regression (LR), Random Forest (RF), Support Vector Machine (SVM), K-Nearest Neighbors (KNN), Gradient Boosting Decision Tree (GBDT), Adaptive Boosting (AdaBoost), Naive Bayes (NB), and Extreme Gradient Boosting (XGBoost).

We employed a 5-fold cross-validated grid search on the training set to optimize hyperparameters. The final hyperparameters were set as follows:

LR: C = 1.0, solver = ‘liblinear’, penalty = ‘l2’, max_iter = 100, tol = 0.01;

RF: n_estimators = 200, max_depth = 50, min_samples_leaf = 10, min_samples_split = 2;

SVM: C = 1, kernel = ‘rbf’, gamma = 0.1;

KNN: n_neighbors = 8, weights = ‘distance’, p = 4;

GBDT: learning_rate = 0.01, n_estimators = 60, max_depth = 2, min_samples_split = 3;

AdaBoost: base_estimator = DecisionTreeClassifier(max_depth = 1), n_estimators = 200, algorithm = ‘SAMME’, learning_rate = 0.01;

NB: var_smoothing = 1e−9;

XGBoost: objective = ‘binary:logistic’, colsample_bytree = 0.7, subsample = 0.6, learning_rate = 0.1, gamma = 2, lambda = 1, max_depth = 3, min_child_weight = 3.

#### External validation

During the external validation phase, the pre-trained optimal models were entirely frozen. No retraining or hyperparameter tuning was performed on the external dataset. The models were directly applied to the external validation cohort to assess their generalizability in a real-world clinical scenario.

#### Model interpretation and visualization

To interpret predictions, we used the SHAP method, implemented via the “shap” library. This approach, based on game theory, clarifies a model’s output by calculating the contribution of each feature to the final prediction. The results were then visualized with SHAP summary plots, including bee swarm and bar plots, to identify the key features driving the model’s decisions.

#### Reliability and stability assessment of feature importance

To validate true associations by examining consistency and dose-response relationships, we employed a “Leave-Top1-Out” validation strategy to examine ordered sets of features. This involved removing the highest-ranking feature identified by the initial SHAP analysis from the dataset, retraining the models on the reduced feature set, and comparing the ranking of the remaining features. This approach assesses whether the feature importance is stable or heavily dependent on a single dominant variable. Additionally, we calculated Spearman’s rank correlation coefficients between the key identified features and the clinical prognosis to validate these associations using a non-model-based statistical approach.

### Quantification and statistical analysis

Statistical analyses were conducted using SPSS version 29.0, R version 4.2.3 and Python 3.12. Normality was checked for continuous variables. Independent *t*-tests were used for normally distributed data (mean ± SD); Mann-Whitney *U* tests for non-normally distributed data (median, IQR); *χ*^*2*^ tests for categorical data (%). Univariate and multivariate Cox regression (forward stepwise) identified independent risk factors. Survival curves were plotted using the Kaplan-Meier method with the “survival” and “survminer” packages. Results were reported as Hazard Ratios (HR) with 95% Confidence Intervals (CI). A *p*-value <0.05 was considered statistically significant.

Models were trained on the training set and evaluated on both the independent internal and external validation sets. Performance metrics were calculated using the following equations: *Accuracy* = (*TP* + *TN*)/(*TP* + *TN* + *FP* + *FN*); *Precision* = *TP*/(*TP* + *FP*); *Recall* = *TP*/(*TP* + *FN*); *F*1-*score* = 2×*Precision*×*Recall*/(*Precision*+*Recall*). Where TP (True Positive) refers to poor prognosis patients correctly identified; TN (True Negative) refers to good prognosis patients correctly identified; FP (False Positive) refers to good prognosis patients incorrectly predicted as poor; and FN (False Negative) refers to poor prognosis patients incorrectly predicted as good. Additional evaluations included Confusion Matrices, ROC curves with AUC calculated using “Matplotlib”, and DCA. To rigorously assess the statistical reliability of the models’ discriminative power, 95% CIs for the AUCs in both internal and external validation sets were calculated using a stratified bootstrapping approach with 1,000 resamples.
