# Supplementary material for: Predicting and explaining poor prognosis in diabetic kidney disease using SHAP-based interpretable machine learning
Source: iScience. 2026 Jun 5;29(6):116280. doi: 10.1016/j.isci.2026.116280 (PMC13264261; doi:10.1016/j.isci.2026.116280)
Supplement: Document S1. Tables S1 and S2 [file mmc1.pdf]

iScience, Volume *29*

Supplemental information

Predicting and explaining poor prognosis in diabetic kidney disease using SHAP-based interpretable machine learning

Man Qian, Lin Li, Yanli Cheng, Yue Hou, Shaojie Fu, and Zhonggao Xu

Table S1. Baseline characteristics of the model development cohort and the external validation cohort.

Variables	Model Development (*n*=168)	External Validation (*n*=58)	*P*-value
**Demographics**			
Gender			0.626
Male	96 (57.1%)	31 (53.4%)	
Female	72 (42.9%)	27 (46.6%)	
Age (y)	53.0 (44.0, 60.0)	55.5 (42.0, 63.0)	0.250
**Medical conditions**			
Age at diabetes diagnosis (y)	39.0 (32.0, 49.0)	44.5 (34.0, 51.8)	0.132
Duration of diabetes (y)	10.0 (5.0, 15.0)	10.0 (4.8, 15.0)	0.526
Hypertension			<0.001
No	11 (6.5%)	12 (20.7%)	
Grade 1	3 (1.8%)	2 (3.4%)	
Grade 2	35 (20.8%)	9 (15.5%)	
Grade 3	108 (64.3%)	35 (60.3%)	
Age at hypertension diagnosis (y)	47.0 (39.0, 55.0)	48.9 (39.8, 57.5)	0.508
Duration of hypertension (y)	2.0 (0.3, 6.0)	1.0 (0.4, 6.3)	0.685
**Model Predictors**			
Pathology			0.029
Early	73 (43.5%)	22 (37.9%)	
Nodular sclerosing	85 (50.6%)	20 (34.5%)	
Diffuse sclerosing	10 (6.0%)	16 (27.6%)	
Scr (μmol/L)	124.8 (90.4, 181.0)	111.7 (93.4, 155.1)	0.379
eGFR (ml/min/1.73m²)	52.1 (31.5, 71.8)	52.8 (35.3, 69.6)	0.648
Hb (g/L)	112 (97, 130)	116 (102, 134)	0.211
Alb (g/L)	28.9 ±6.5	28.6 ±5.8	0.756
MAU (mg/24h)	3987.2 (2077.2, 6373.7)	3425.4 (1175.9, 5365.7)	0.127
24hU α1 microglobulin (mg/24h)	55.5 (28.4, 95.3)	71.0 (37.2, 91.2)	0.345
C3 (g/L)	1.10 (0.97, 1.21)	1.18 (1.06, 1.36)	0.008
URBC (/HPF)	3.1 (1.5, 9.2)	3.0 (2.0, 8.8)	0.883
**Follow-up results**			
Composite endpoint			0.951
Yes	79 (47.0%)	27 (46.6%)	
No	89 (53.0%)	31 (53.4%)	
Time (m)	17.7 (11.5, 30.2)	18.8 (14.6, 30.5)	0.207

Table S2. Model performance on the external validation cohort.

Model	Accuracy	Precision	Recall	F1-score	AUC (95% *CI*)
LR	0.759	0.724	0.778	0.750	0.829 (0.720, 0.928)
RF	0.724	0.704	0.704	0.704	0.824 (0.712, 0.931)
SVM	0.793	0.742	0.852	0.793	0.833 (0.714, 0.935)
NB	0.724	0.704	0.704	0.704	0.834 (0.713, 0.929)
